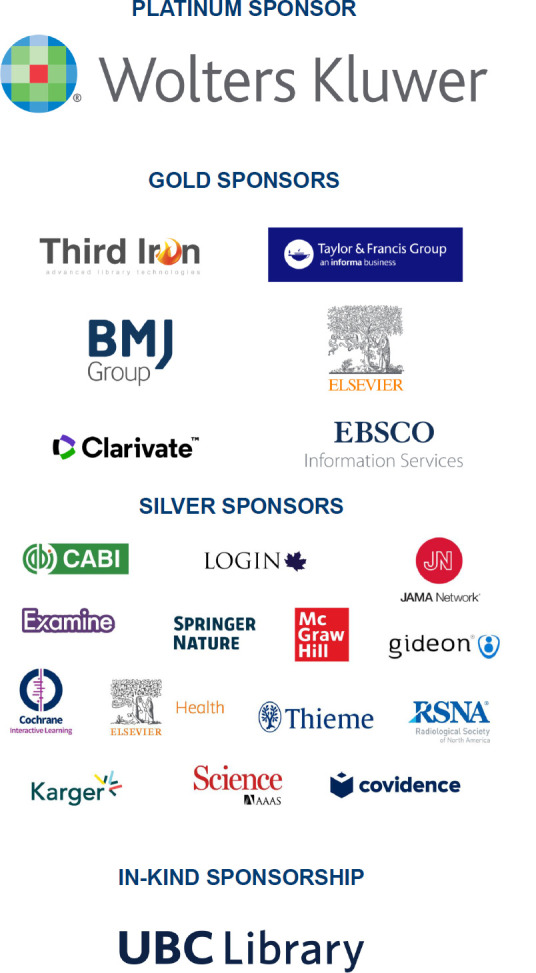# CHLA 2025 Conference Sponsors / ABSC Congrès 2025 Partenaires Industriels

**Published:** 2025-08-01

Thank you to the 2025 conference sponsors!

Your generous and steadfast support for the CHLA/ABSC is appreciated.